# Antagonism of proteasome inhibitor-induced heme oxygenase-1 expression by PINK1 mutation

**DOI:** 10.1371/journal.pone.0183076

**Published:** 2017-08-14

**Authors:** Xiang-Jun Sheng, Hunag-Ju Tu, Wei-Lin Chien, Kai-Hsiang Kang, Dai-Hua Lu, Horng-Huei Liou, Ming-Jen Lee, Wen-Mei Fu

**Affiliations:** 1 Pharmacological Institute, College of Medicine, National Taiwan University, Taipei, Taiwan; 2 Department of Neurology, National Taiwan University Hospital, Taipei, Taiwan; Faculty of Biochemistry, Biophysics and Biotechnology, Jagiellonian University, POLAND

## Abstract

PTEN-induced putative kinase 1 (PINK1) is an integral protein in the mitochondrial membrane and maintains mitochondrial fidelity. Pathogenic mutations in *PINK1* have been identified as a cause of early-onset autosomal recessive familial Parkinson’s disease (PD). The ubiquitin proteasome pathway is associated with neurodegenerative diseases. In this study, we investigated whether mutations of PINK1 affects the cellular stress response following proteasome inhibition. Administration of MG132, a peptide aldehyde proteasome inhibitor, significantly increased the expression of heme oxygenase-1 (HO-1) in rat dopaminergic neurons in the substantia nigra and in the SH-SY5Y neuronal cell line. The induction of HO-1 expression by proteasome inhibition was reduced in PINK1 G309D mutant cells. MG132 increased the levels of HO-1 through the Akt, p38, and Nrf2 signaling pathways. Compared with the cells expressing WT-PINK1, the phosphorylation of Akt and p38 was lower in those cells expressing the PINK1 G309D mutant, which resulted in the inhibition of the nuclear translocation of Nrf2. Furthermore, MG132-induced neuronal death was enhanced by the PINK1 G309D mutation. In this study, we demonstrated that the G309D mutation impairs the neuroprotective function of PINK1 following proteasome inhibition, which may be related to the pathogenesis of PD.

## Introduction

Parkinson’s disease (PD) is a debilitating neurodegenerative movement disorder [1, 2]. The neuropathological changes of PD are characterized by preferential degeneration of dopaminergic neurons in the substantia nigra pars compacta and by the intracellular accumulation of proteinaceous inclusions in the surviving dopaminergic neurons [1, 3, 4]. Although the etiology of PD is still not completely clear, studies have proposed that mitochondrial dysfunction, oxidative stress, ubiquitin proteasome system (UPS) impairment, and abnormal protein accumulation are associated with the pathogenesis of PD [5–7]. Epidemiological studies have revealed that approximately 5%-10% PD cases are hereditary [8]. The estimated prevalence of *PINK1* mutations in different ethnicities is 1%-8% of familial or early-onset PD, and *PINK1* gene mutations represent the second most frequent cause after *PARKIN* [9, 10]. PINK1 consists of 581 amino acids and contains an N-terminal mitochondria-targeting sequence, followed by a transmembrane domain, a serine threonine kinase domain, and a regulatory C-terminal domain [11]. The first reported *PINK1* mutation, G309D, was identified in Spanish patients. Wild-type *PINK1*(WT-PINK1), but not *PINK1* with the G309D mutation, can protect cells against the loss of mitochondrial function caused by stress [12]. The majority of PD-linked PINK1 mutations are localized within the kinase domain of PINK1 [11]. The kinase activity of PINK1 has been hypothesized to be required for PINK1 to exert its neuroprotective effects in dopaminergic neurons [11].

Proteotoxic stress caused by protein misfolding is a hallmark of neurodegeneration and is further exacerbated in PD by the loss of proteasome activity [13]. The UPS is responsible for the clearance of abnormal proteins including unfolded or misfolded, mutant and damaged (e.g., by oxidative injury) proteins [1, 14]. Impairment of UPS function has been regarded as a crucial mechanism involved in the pathogenesis of PD [7, 15]. When the UPS function is inhibited, the accumulation and aggregation of abnormal proteins may occur and cause neurotoxicity [16].

Heme oxygenase-1 (HO-1) has long been regarded as a potent antioxidant [17]. HO-1 has also been recognized as a dynamic sensors of cellular oxidative stress because it can be strongly induced by cellular stress and various oxidative challenges [17–19]. HO-1 has been reported to potentially exhibit cytoprotective activities including antioxidant and anti-inflammatory effects and inhibition of cellular apoptosis [20–22]. Expression of HO-1 is modulated by transcriptional regulation and nuclear factor-E2-related factor 2 (Nrf2) is a key transcription factor for the induction of HO-1 expression [23]. In this study, we investigated the effect of PINK1 mutation on HO-1 expression following proteasome inhibition.

## Materials and methods

### Reagents

The pAcGFP-Hyg-N1/PINK1 mutant expression vector harboring the WT-PINK1or PINK1 G309D mutant was derived from the laboratory of Dr. Ming-Jen Lee. Peptide aldehyde proteasome inhibitor MG132 (Z-Leu-Leu-Leu-al), free radical scavenger N-acetyl-L-cysteine (NAC), 2'-amino-3'-methoxyflavone (PD98059), 2-(4-Morpholinyl)-8-phenyl-4H-1-benzopyran-4-one (LY294002), 4-(4-fluorophenyl)-2-(4-methylsulfinylphenyl)-5-(4-pyridyl)1H-imidazole (SB203580), 3-(4,5-dimethylthiazole-2yl)-2,5-diphenyltetrazolum bromide (MTT), 2’,7’-dichlorofluorescein (DCF) and hygromycin were purchased from Sigma-Aldrich (MO, U.S.A.).

Rabbit anti-HO-1 and anti-Nrf2 antibodies were from Abcam (MA, U.S.A.). Mouse anti-α-tubulin was from Novus (Littleton, CO). Mouse anti- tyrosine hydroxylase (TH) was from Calbiochem (San Diego, CA). Mouse anti-C23, anti-p-p38, anti-p-ERK, rabbit anti-ERK2 antibodies were from Santa Cruz Biotechnology (Santa Cruz, CA). Mouse anti-catalase was from Sigma-Aldrich. Mouse anti-β-actin, rabbit anti-p-Akt, anti-Akt, and anti-p38 MAPK antibodies were from Cell Signaling Technology (Beverly, MA).

### Cell culture

SH-SY5Y cells, a human dopaminergic neuroblastoma cell line, were maintained in F12/ MEM (1:1) (GIBCO, NY, U.S.A.) and supplemented with 10% heat-inactivated fetal bovine serum (Biological Industries, Israel), 1% non-essential amino acid (Biological Industries, Israel) and 1% penicillin-streptomycin (5000 units/mL, 5000 μg/mL) (GIBCO, NY, U.S.A.). Cells were cultured in a humidified incubator with 5% CO_2_ at 37°C.

### Selection of stable clone expressing PINK1 G309D mutant

The pAcGFP-Hyg-N1/PINK1 mutant expression vectors harboring the PINK1 G309D mutant were constructed. The plasmids were transfected into the SH-SY5Y cells by using Lipofectamine 2000 (Invitrogen, CA, U.S.A.). Hygromycin (150 μg/mL; Sigma-Aldrich) was added to the cultures for selection of stable clones. After 4–6 weeks, the stable clones containing SH-SY5Y/PINK1 G309D were isolated. For the characterization of stable clone, the genomic DNA was extracted using the DNA Purification Kit (Promega, WI, U.S.A.). The cell genomic DNA was subjected to PCR with a primer set, PINK1_818–835 (forward: ACATCATCCGGGTTCTCC and PINK1_1118–1101 (reverse: TCCAGCTCCACAAGGATG). The size of the amplicons in genomic DNA is 1188 bp (sequence from http://www.ensembl.org/Homo_sapiens/Gene/Sequence?g=ENSG00000158828;r=1:20959948-20978004) and the size for cDNA clone is 301 bp.

### Animals

The experiments were carried out in accordance with the guidelines of the National Institutes of Health in the USA regarding the care and use of animals for experimental procedures. Male Wistar rats weighing approximately 300 g, were purchased from LASCO Inc. (Taipei, Taiwan). Rats were housed in groups of 3 per cage under a controlled 12-hour light/dark cycle (light from 0800 to 2000 hours), at 23 ± 1°C and 55 ± 5% humidity, with free access to food and water. All experiments were performed in accordance with Taiwanese animal protection laws and approved by the Institutional Animal Care and Use Committee of National Taiwan University (IACUC 20110175).

### Intra-substantia nigra injection of MG132

Rats received unilateral intra-substantia nigra injection of saline or MG132 (9.5 ng) under general anesthesia (0.8–1.5% isoflurane in 30% oxygen with air). The stereotaxic coordinates for substantia nigra were laternal +2.0 mm, antero-posterior from the bregma point -5.3 mm and dorsi-ventral +7.8 mm [24]. The solution was injected into the substantia nigra with 10 μl Hamilton syringe coupled to a motorized injector (Stoelting, Wood Dale, IL) at a rate of 0.2 μl/min and the needle was left in situ for at least 5 min after injection. After surgery, the rats were kept in the same cages for recovery. Rats expressing infection signs were excluded from the study. The rats were sacrificed using overdose isoflurane at 24 h after MG-132 injection. For double immunofluorescent labelling studies, the substantia nigra sections were stained with two primary antibodies, mouse anti- TH (1:1000) and rabbit anti-HO-1 (1:500), then with Alexa 488 (excitation 495 nm, emission 519 nm) or Alexa 546 (excitation 556 nm, emission 573 nm)-conjugated goat anti-rabbit or anti-mouse secondary antibody (1:500; Invitrogen, CA, USA). DAPI (1 μg/ml) was used to stain nucleus and the fluorescent images were obtained using a confocal microscope (model SP5 LAS; Leica, Heidelberg, Germany).

### Measurement of reactive oxygen species (ROS)

The level of cytosolic ROS was measured by using 2’,7’-dichlorofluorescein (DCF). Briefly, SH-SY5Y cells were grown in a 24-well plate and replaced the media without phenol red. Cells were incubated with MG132 (1 μM) and DCFH-DA for 4 h. DCFH-DA was initially non-fluorescent and was converted by oxidation to the fluorescent molecule DCFH, which was measured at 492 nm excitation and 520 nm emission wavelength at different time intervals using a Microplate fluorometer (Spectra Max Gemini XS, Molecular Devices, Sunnyvale, CA).

### Western blot

The whole cell lysates were obtained on ice by using RIPA buffer (150 mM NaCl, 50 mM Tris-HCl, 0.05 mM EGTA, 1.0% NP-40, 0.25% deoxycholic acid, pH7.4) and Halt Protease and Phosphatase Inhibitor Cocktail (Thermo Scientific, IL, U.S.A.). After sonication and centrifugation at 14,500 r.p.m. for 30 min at 4°C, the supernatant was collected and the protein concentrations were measured using BCA Protein Assay (Thermo Scientific, IL, U.S.A.).

Protein samples were boiled for 5 min at 95°C. For each sample, 20–35 μg of total protein along with a protein ladder (Thermo Scientific, IL, U.S.A.) were separated on sodium dodecyl sulfate/polyacrylamide gel electrophoresis and then transferred onto nitrocellulose membranes (Invitrogen, CA, U.S.A.). Membranes were blocked in phosphate buffered saline containing 0.1% Tween 20 (PBST) with 10% dry skim milk for 1 h at room temperature. The membranes were then incubated at 4°C overnight with appropriate primary antibodies diluted in PBST. After incubation with primary antibody, membranes were washed in PBST three times and then incubated for 1 h at room temperature with suitable peroxidase-conjugated secondary antibody in PBST. After washing, the proteins of interest were detected by enhanced chemiluminescence (Millipore, MA, U.S.A.) using Kodak X-OMAT LS film (Eastman Kodak, Rochester, NY, USA) or by a UVP imaging system with LabWorks Software (Upland, CA, U.S.A.).

### Quantitative real time-PCR

Total RNA were extracted from cells using TRIzol reagent (MDBio Inc., Taipei, Taiwan) and cNDA synthesized using the M-MLV Reverse Transcriptase kit (Promega, WI, U.S.A.), according to the manufacturer’s instructions. Quantitative real time-PCR was executed using Fast SYBR Green Master Mix kit (Applied Biosystems, CA, U.S.A.) and analyzed with a model StepOnePlus Real time-PCR system (Applied Biosystems, CA, U.S.A.). Human HO-1 and Nrf2 was amplified with gene-specific primers (HO-1 forward primer: 5’- AGAGCTGCACCGCAAGGCTG -3’, reverse primer: 5’- ACCAGCAGCTCGGGCTCTGT -3’; Nrf2 forward primer: 5’- GCCTTCCGTGTC- CCCACTGC -3’, reverse primer: 5’- CAATGCCAGCCCCAGCGTCA -3’) [25]. The values of HO-1 and Nrf2 expression were normalized with the β-actin copy number (β-actin forward primer: 5’-CATGTTTGAGACCTTCAACAC-3’, reverse primer: 5’-CCAGGAAGGAAGGCTGGAA-3’). After PCR amplification, the melting curve must be determined for all samples, the single peak represents the product specificity. Analysis of the standard curve with each primer suggests that the threshold was in the linear range for the used template amount. The mRNA expression of target gene can be calculated by the cycle number which the transcript was detected (C_t_). The quantification was expressed as a ratio of 2^ΔCt^ of target gene to 2^ΔCt^ of internal control.

### Cell viability evaluation using MTT assay

Cell viability was determined by a MTT assay. Cells were seeded in 24-well plate. After treatment, the growth media were exchanged by MTT solution (0.5 mg/mL) and incubated for 45 min at 37°C. Subsequently, the medium containing MTT was discarded and dimethylsulfoxide was added for cell lysis. Cell viability was determined photometrically at the absorbance wavelength of 570 nm (reference: 630 nm).

### Isolation of nucleus fraction

For preparation of nuclear extracts, cells were cultured, and appropriate treatment was performed in 10 cm dishes. Cytoplasmic and nuclear extracts were separated by using NE-PER Nuclear and cytoplasmic extraction reagents (Thermo Scientific, IL, U.S.A.) according to the manufacturer’s protocol.

### Small interfering RNA (siRNA) transfection

Cells were transfected with negative control siRNA or Nrf2-specific siRNA using Lipofectamine 2000 (Invitrogen) for 48 h according to the manufacturer’s instructions. After transfection, cells were incubated with MG132 for another 24 h. The cells were then subjected to Western blotting analysis.

### Immunofluorescent staining of cultured cells

For double-immunolabeling studies, SH-SY5Y and SH-SY5Y/PINK1 G309D cells were exposed to MG132 for 6 hours. After washing with PBS, cells were fixed with 4% paraformaldehyde in PBS for 15 min, blocked with PBS containing 0.1% Triton X-100 and 4% bovine serum albumin for 1 h, and then immunostained for overnight at 4°C with primary rabbit antibody against Nrf2 (1: 250; Abcam). Cells were washed three times with PBS, followed by incubation for 1 hour with Alexa-488 conjugated goat anti-rabbit secondary antibody (1: 500; Invitrogen) and then with DAPI (0.5 μg/mL) for 10 min. The images were obtained with fluorescent microscope (Zeiss axio imager. A1; Carl Zeiss, Germany).

### Data analysis

Results were represented as mean± S.E.M of at least three experiments. The statistical significance was assessed by Student’s t test. The difference is significant when *p < 0.05.

## Results

### Upregulation of HO-1 following proteasome inhibition by MG132 in rat substantia nigra

**Fig 1:**
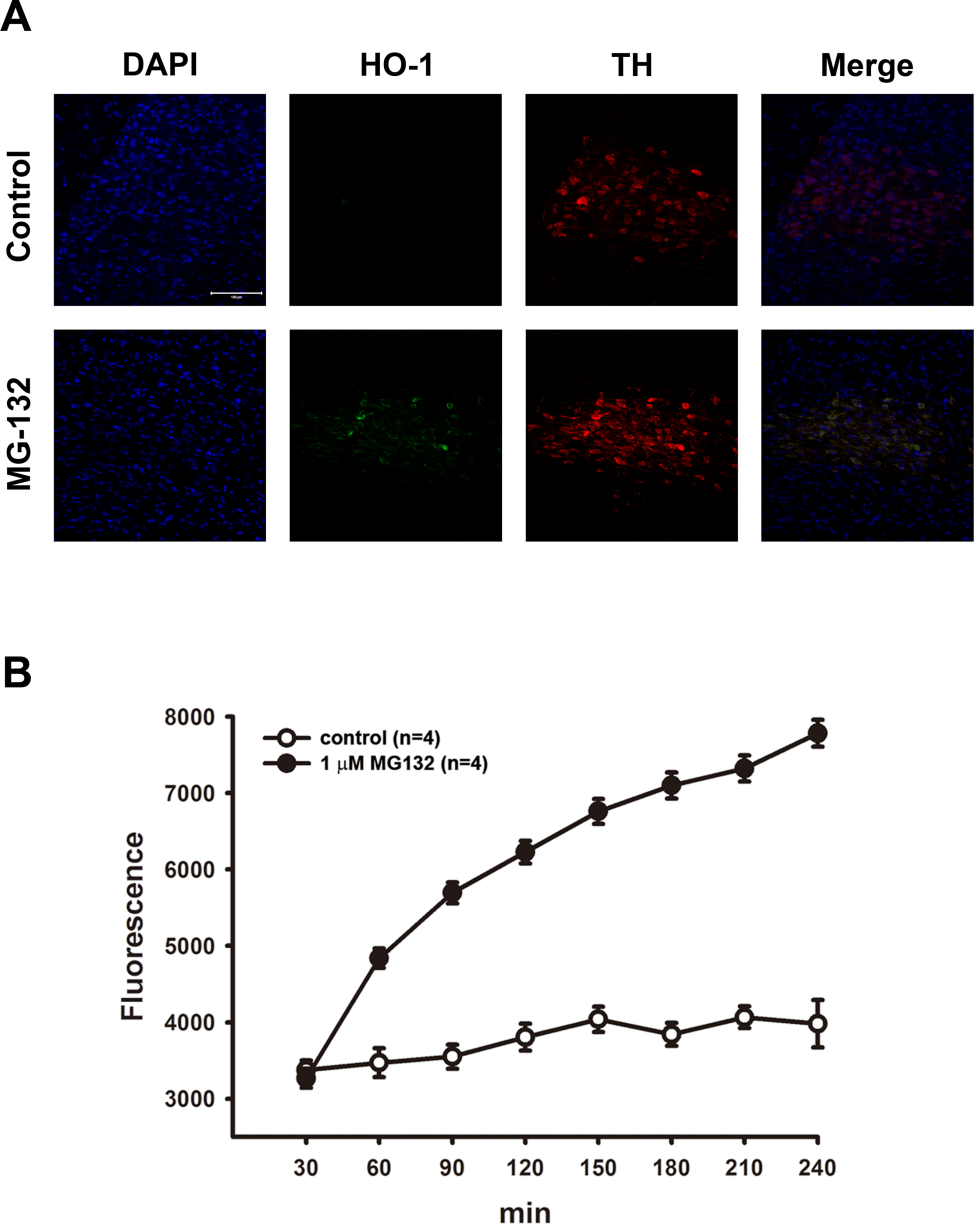
Upregulation of heme oxygenase-1 (HO-1) following proteasome inhibition by MG132 in the substantia nigra of rat. (A) MG132 (9.5 ng) was locally injected into the substantia nigra of rats, and the rats were sacrificed 24 h later. Immunofluorescent staining shows that HO-1 expression increased following MG132 treatment and HO-1 occurred mostly in TH (+) neurons. Scale bar: 100 μm. (B) Treatment with MG132 (1 μM) in cultured SH-SY5Y cells. Levels of ROS were analyzed using a DCF fluorescence assay. Note that the generation of ROS increased time-dependently after MG132-treatment. Results are expressed as percentage of wild-type control (mean ± S.E.M.) (n = 4).

Impairment of UPS function has been regarded as one of the crucial mechanisms in PD pathogenesis [7, 15]. Moreover, proteasome inhibition has been reported to up-regulate the heat shock protein system to prevent cell damage [26]. We first examined whether MG132 treatment increases the expression of HO-1. Rats receiving a single intra-substantia nigra injection of MG132 exhibited a large number of HO-1 immunopositive cells in the substantia nigra 24 h after receiving the MG132 injection (Fig 1A). Cells were stained with DAPI, HO-1 and TH, which showed that the expression of HO-1 was co-localized with TH (+) neurons, thus indicating that MG132-treatment up-regulated HO-1 expression in dopaminergic neurons.

Various proteasome inhibitors, including MG132, have been demonstrated to stimulate subsequent cell signaling through the induction of ROS [27, 28], Here, we examined whether treatment with MG132 increased production of ROS in cultured neuronal cells. After treatment with MG-132, the production of ROS increased in a time-dependent manner in SH-SY5Y cells (Fig 1B).

### Transfection of PINK1 G309D mutant inhibits MG132-induced expression of HO-1 in SH-SY5Y cells

**Fig 2:**
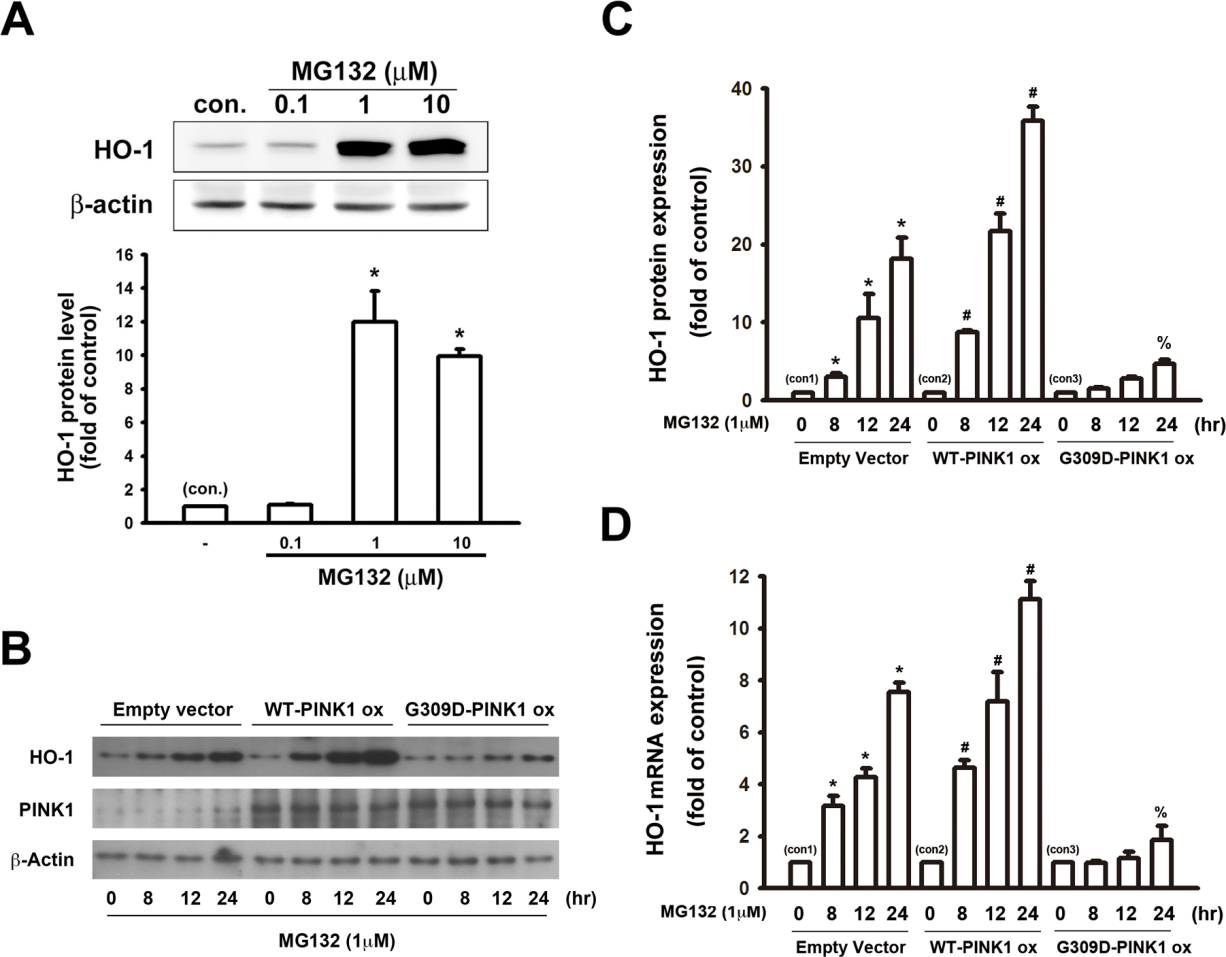
PINK1 G309D mutation inhibits MG132-induced expression of HO-1 in SH-SY5Y cells. (A) SH-SY5Y cells were treated with different concentrations of MG132 for 24 h. Note that MG132 concentration-dependently enhanced HO-1 protein expression. The representative blots are illustrated in the upper panel and the fold-increases measured relative to controls are shown in the lower panel. (B) Empty vector-transfected cells, WT-PINK1 cells and PINK1 G309D mutant cells were treated with MG132 (1 μM) for various time intervals. Note that MG132 time-dependently increased HO-1 protein levels in wild-type cells and overexpression of WT-PINK1 enhanced HO-1 production, whereas the level of HO-1 was impaired in PINK1 G309D mutant cells. The quantitative results are shown in (C). (D) The HO-1 mRNA levels were analyzed by real time-PCR after MG-132 treatment. Note that of HO-1 mRNA was time-dependently upregulated by MG132. In comparison with empty vector-transfected cells, the regulation of HO-1 mRNA levels induced by MG132 was potentiated in WT-PINK1 cells and inhibited by the expression of the recombinant PINK1 G309D mutant. *p < 0.05 compared with empty vector-transfected control cells (con 1); #p < 0.05 compared with the corresponding MG132-treated empty vector-transfected cells (con 2). %p < 0.05 compared with the correspondening MG132-treated empty vector-transfected cells (con 3). (ox: overexpression).

Cultured SH-SY5Y neuronal cells were treated with different concentrations of MG132 for 24 hours. A western blot analysis indicated that treatment of MG132 enhanced the protein expression of HO-1 (Fig 2A). We further investigated whether PINK1 G309D mutation affected the induction of HO-1 following proteasome inhibition. Empty vector-transfected cells and PINK1 G309D mutant cells were treated with MG132 (1μM) for various time intervals, and WT-PINK1 cells were used as a positive control group. MG132 time-dependently increased the expression of HO-1 in the control cells. Moreover, the levels of HO-1were enhanced in cells transfected with WT-PINK1, whereas the G309D mutant PINK1 protein exhibited weakened ability to induce HO-1 expression (Fig 2 B). We further investigated whether MG132-induced HO-1 protein expression was associated with the elevation of HO-1 mRNA expression. Real time-PCR analysis indicated that MG132 time-dependently increased HO-1 mRNA levels. The HO-1 mRNA levels further increased in the cells with WT-PINK1 overexpression and were abrogated by the PINK1 G309D mutation (Fig 2D).

### Effect of PINK1 G309D mutation on Nrf2-antioxidant response element pathway following proteasome inhibition

**Fig 3:**
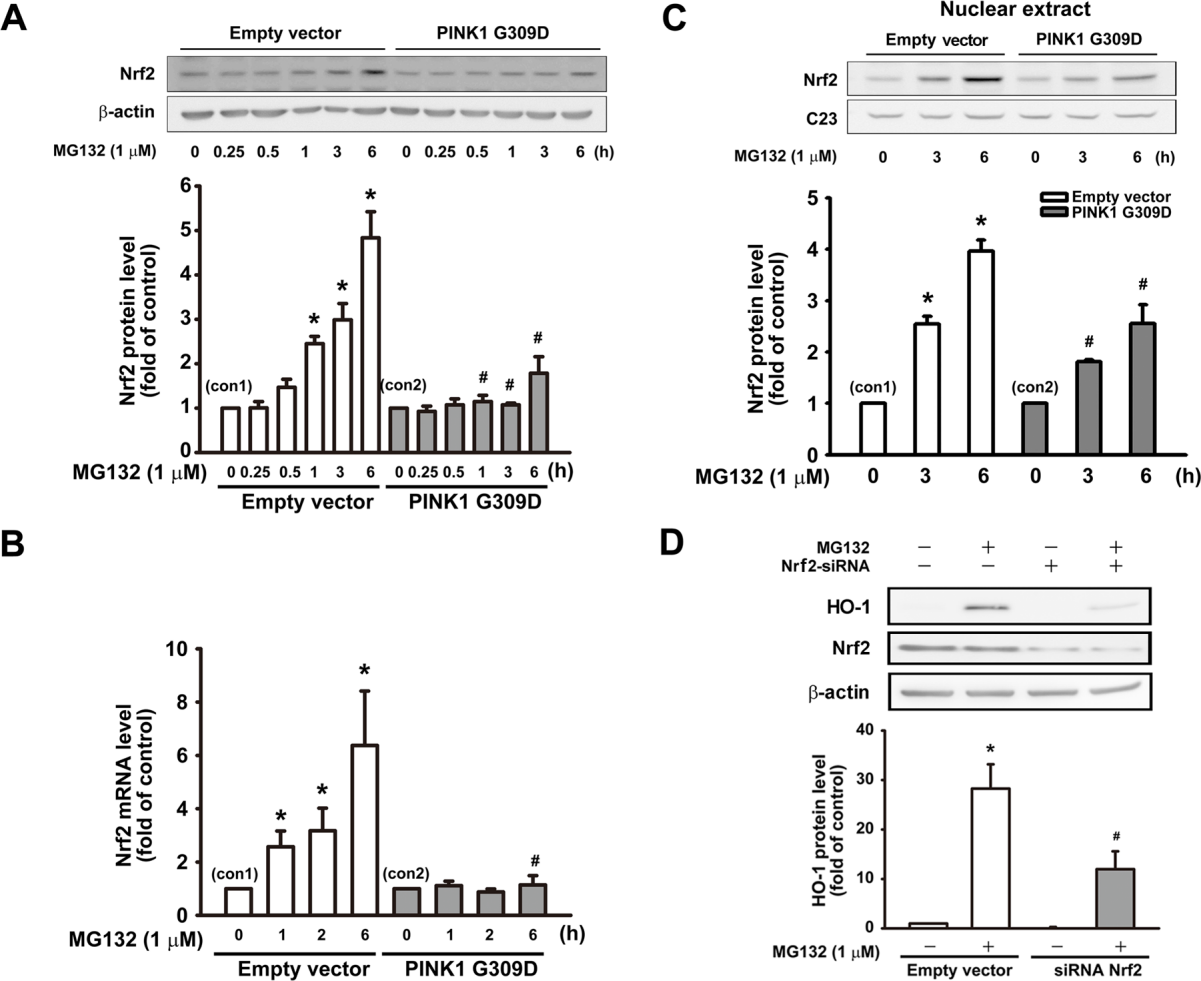
PINK1 G309D mutation inhibits MG132-induced Nrf2 expression and nuclear translocation of Nrf2 in SH-SY5Y cells. Empty vector-transfected cells and PINK1 G309D mutant cells were treated with MG132 (1 μM) for various time intervals. (A) MG132 time-dependently increased the expression of Nrf2, which was inhibited by the expression of the recombinant PINK1 G309D mutant. (B) The Nrf2 mRNA levels were analyzed by real time-PCR. Note that PINK1 G309D mutant cells inhibited Nrf2 mRNA upregulation following MG132 treatment. (C) Empty vector-transfected cells and PINK1 G309D mutant cells were treated with MG132 (1 μM) for 3 and 6 h, and the nuclear extracts were then obtained to evaluate the Nrf2 expression in nucleus. It showed MG132 time-dependently increased the nuclear translocation of Nrf2, which was inhibited by the PINK1 G309D mutation. C23 served as a nuclear internal control in the nucleus. (D) Cells were transfected with scramble or siRNA against Nrf2 and then treated with vehicle or MG132. Note that MG132-induced upregulation of HO-1 protein was antagonized by the knockdown of Nrf2. Data are presented as multiples of respective controls (mean ± S.E.M) (n = 4). *p < 0.05 compared with the empty vector or scramble RNA -transfected control cells; #p < 0.05 compared with the corresponding MG132-treated control.

The induction of HO-1 is mainly connected with the Nrf2-antioxidant response element pathway [21, 23]. Nrf2 plays a critical role in the inducible expression of antioxidant genes [29, 30]. As mentioned previously, MG132 increased HO-1 expression, which was abrogated by the PINK1 G309D mutation. For elucidating the underlying mechanisms behind the induction of antioxidative enzymes by proteasome inhibition, we further investigated whether MG132 regulated the expression of Nrf2. It showed that MG132 time-dependently increased the Nrf2 protein level, which was inhibited by PINK1 G309D mutation in the SH-SY5Y neuronal cells (Fig 3A). We also examined whether MG132-induced Nrf2 protein expression was associated with the increase in Nrf2 RNA expression. Real time-PCR showed that MG132 treatment also time-dependently increased Nrf2 mRNA expression, which was antagonized by the PINK1 G309D mutation (Fig 3B).

**Fig 4:**
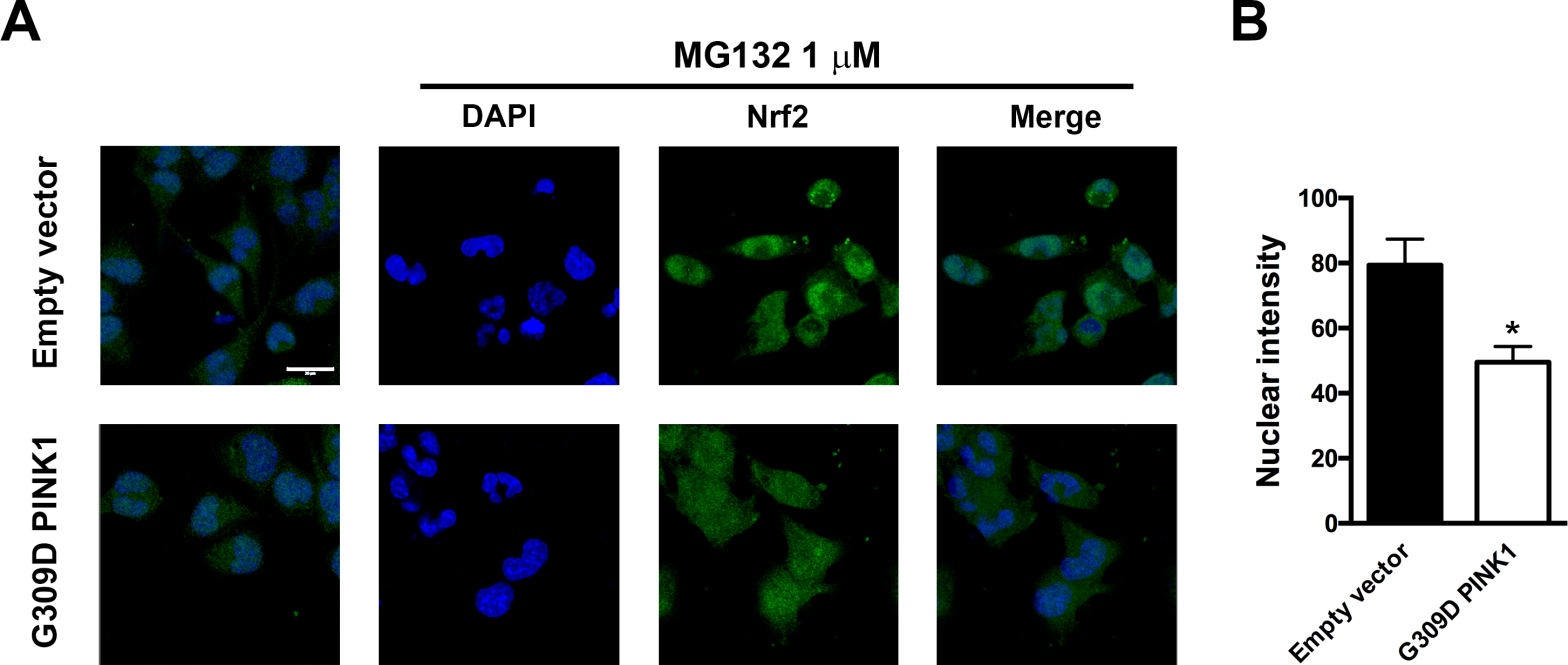
MG132-induced Nrf2 accumulation and nuclear translocation in SH-SY5Y cells is inhibited by the expression of PINK1 G309D mutant. Empty vector-transfected cells and PINK1 G309D mutant cells were treated with MG132 (1 μM) for 6 h. (A) The expression of PINK1 G309D mutant inhibited the nuclear translocation and accumulation of Nrf2 following MG132 treatment. The nucleus is depicted in blue (DAPI) and Nrf2 is presented in green (AlexaFluor488). Scale bar: 20 μm. (B) Quantification of nuclear intensity.

Nrf2 has been reported to bind to Keap1 in the cytoplasm under basal conditions, and it translocates into the nucleus and then binds to the antioxidant response element of target genes upon stimulation and activation [30, 31]. We then further examined whether MG132 treatment enhanced the nuclear translocation of Nrf2. Empty vector-transfected cells and G309D mutant cells were treated with MG132 (1 μM) for 3 and 6 h. MG132 increased the nuclear translocation of Nrf2 in cells with WT-PINK1, whereas the expression of the recombinant PINK1 G309D mutant inhibited the nuclear translocation of Nrf2 (Fig 3C). To confirm the involvement of Nrf2 in HO-1 induction, Nrf2 siRNA was used to knockdown Nrf2 in the SH-SY5Y cells. Following treatment with Nrf2 siRNA for 48 h, MG132 was added for another 24 h. Nrf2 siRNA treatment significantly reduced the upregulation of HO-1 induced by MG132 (Fig 3D). Furthermore, immunofluorescent staining revealed that the treatment of MG132 (1 μM) for 6 h enhanced the nuclear expression of Nrf2 in the empty vector-transfected cells after proteasome inhibition, which was inhibited by PINK1 G309D mutation (Fig 4).

### Effect of PINK1 G309D mutation on the signaling pathways involved in MG132-induced HO-1 expression

**Fig 5:**
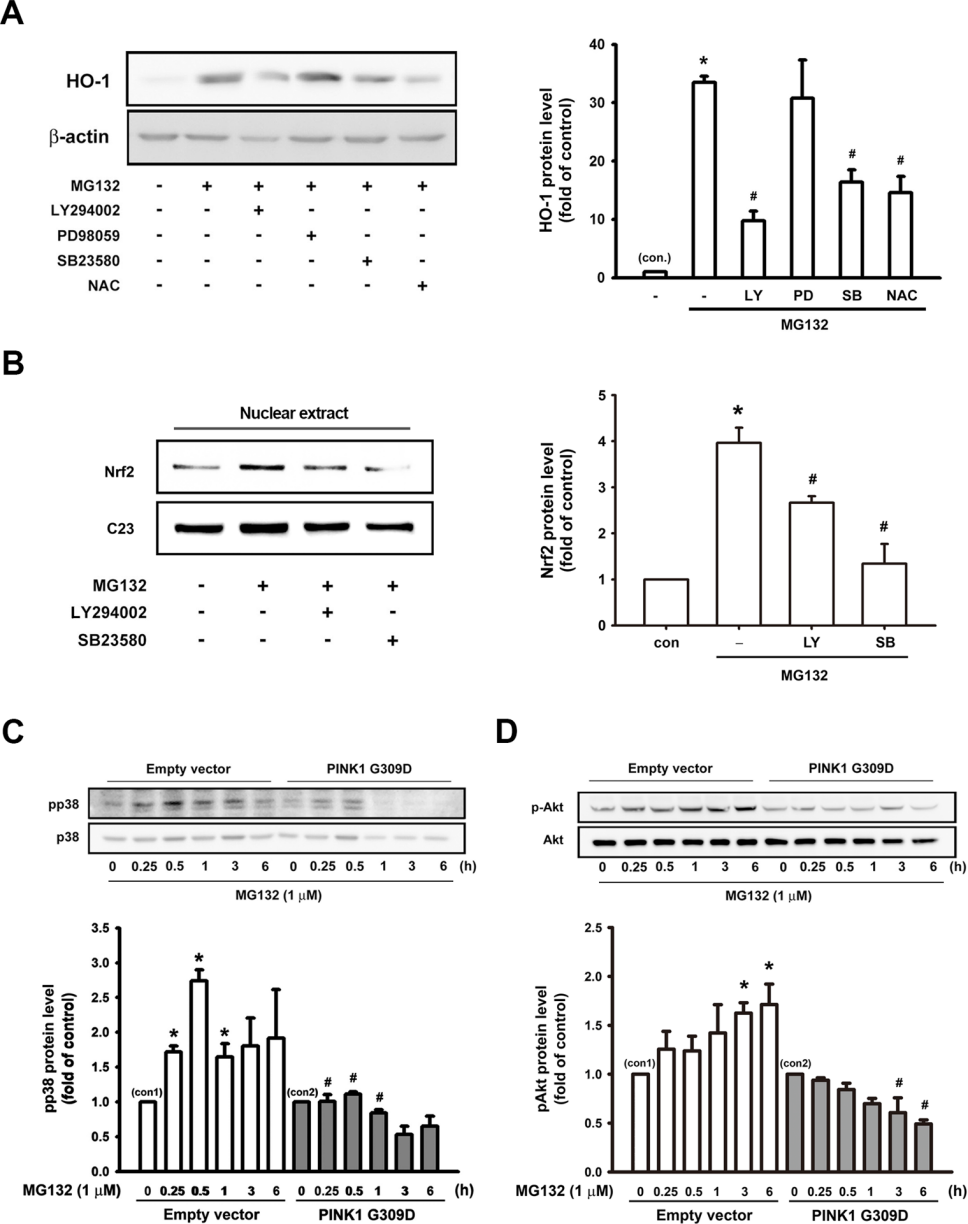
Effects of PINK1 G309D mutant on the signaling pathways involved in MG132-induced HO-1 expression and Nrf2 activation in SH-SY5Y cells. (A) SH-SY5Y cells were pretreated for 30 min with LY294002 (20 μM), PD98059 (20 μM), SB23580 (10 μM) or N-Acetyl-L-cysteine (NAC) (10 mM). Subsequently, MG132 (1 μM) was added for 24 h. (B) SH-SY5Y cells were were pre-treated for 30 min with LY294002 (20 μM) or SB23580 (10 μM). Then, MG132 (1 μM) was added for 6 h, and the nuclear extracts were then prepared for the evaluation of Nrf2 protein levels. Note that PI3K/Akt inhibitor, p38 MAPK inhibitor, and NAC significantly antagonized the induction of HO-1 by MG132. Furthermore, PI3K/Akt or p38 MAPK inhibitor inhibited MG132-induced Nrf2 nuclear translocation. MG132 time-dependently increased p38 (C) and Akt (D) phosphorylation and expression of recombinant PINK1 G309D mutant inhibited the phosphorylation of p38 and Akt following MG132 treatment. Results are expressed as multiples of controls (mean ± S.E.M) (n = 3). *p < 0.05 compared with control group (con.); #p < 0.05 compared with MG132 treatment alone.

In order to elucidate the signaling mechanisms underlying the induction of antioxidative enzymes by proteasome inhibition, several pharmacological inhibitors were used to evaluate the effects of MG132 on HO-1 induction. As shown in Fig 5A, the application of 1 μM MG132 increased HO-1 protein expression in the SH-SY5Y cells, which was antagonized by the application of LY294002 (20 μM, a PI3K/Akt inhibitor), SB203580 (10 μM, a p38 MAPK inhibitor) and N-acetyl-L-cysteine (NAC, 10 mM, a free radical scavenger), but not by PD98059 (20 μM, an ERK inhibitor). In addition, Nrf2 nuclear translocation was inhibited by LY294002 and SB203580 (Fig 5B). These results indicated the critical role of the PI3K/Akt and p38 MAPK signaling pathways in MG132-induced HO-1 expression.

The phosphorylation of Akt and p38 MAPK after MG132 treatment was further assessed. MG132 (1 μM) treatment time-dependently increased the phosphorylation of p38 MAPK and Akt, which was antagonized by the PINK1 G309D mutation (Fig 5C and 5D). Inhibitors alone did not affect the basal levels of HO-1 or Nrf2 expression (S2A and S2B Fig). Taken together, these results demonstrated that PINK1 plays a crucial role in HO-1 induction and is a sensor for the activation of p38MAPK, Akt and Nrf2 in the presence of stress.

### Enhancement of MG132-induced neuronal death by PINK1 G309D mutation

**Fig 6:**
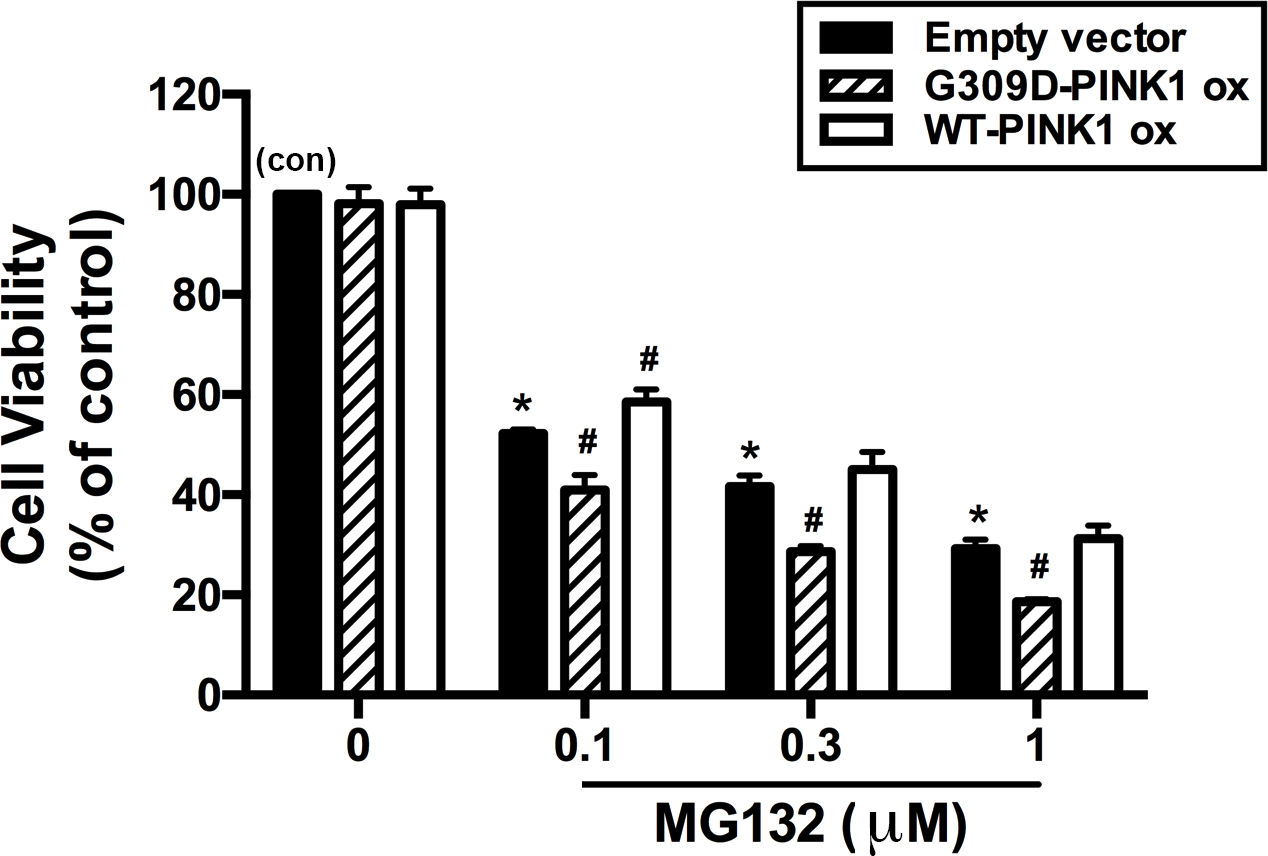
Enhancement of MG132-induced neuronal death by PINK1 G309D mutation. Empty vector-transfected cells and PINK1 G309D mutant cells were treated with different concentrations of MG132 for 24 h. Subsequently, the cell viability was evaluated by MTT assay. Note that transfection of WT-PINK1 or G309D PINK1 alone did not affect cell viability. MG132 concentration-dependently induced cell death, which was enhanced by the PINK1 G309D mutation. Results are presented as percentage of control (mean ± S.E.M) for three experiments performed in triplicate. *p < 0.05 compared with the empty vector-transfected control cells (con); #p < 0.05 compared with the respective empty vector cells in the presence of various concentrations of MG132.

Inhibition of the proteasome activity causes neurotoxicity [16]. To examine whether recombinant PINK1 G309D mutation affects neuronal death following proteasome inhibition in SH-SY5Y cells, cell viability was measured through the MTT assay. As shown in Fig 6, compared with the parental cells, the survival rates were almost identical when either WT-PINK1 or G309D PINK1 was overexpressed in the treated SH-SY5Y cells. Moreover, the SH-SY5Y cells with overexpression of WT-PINK1 resisted the toxicity of 0.1 μM MG132 more effectively than could the parental SH-SY5Y cells. However, G309D PINK1 potentiated cell death in SH-SY5Y cells after proteasome inhibition. These results indicated that the expression of the recombinant PINK1 G309D mutant potentiated cell death following proteasome inhibition.

## Discussion

**Fig 7:**
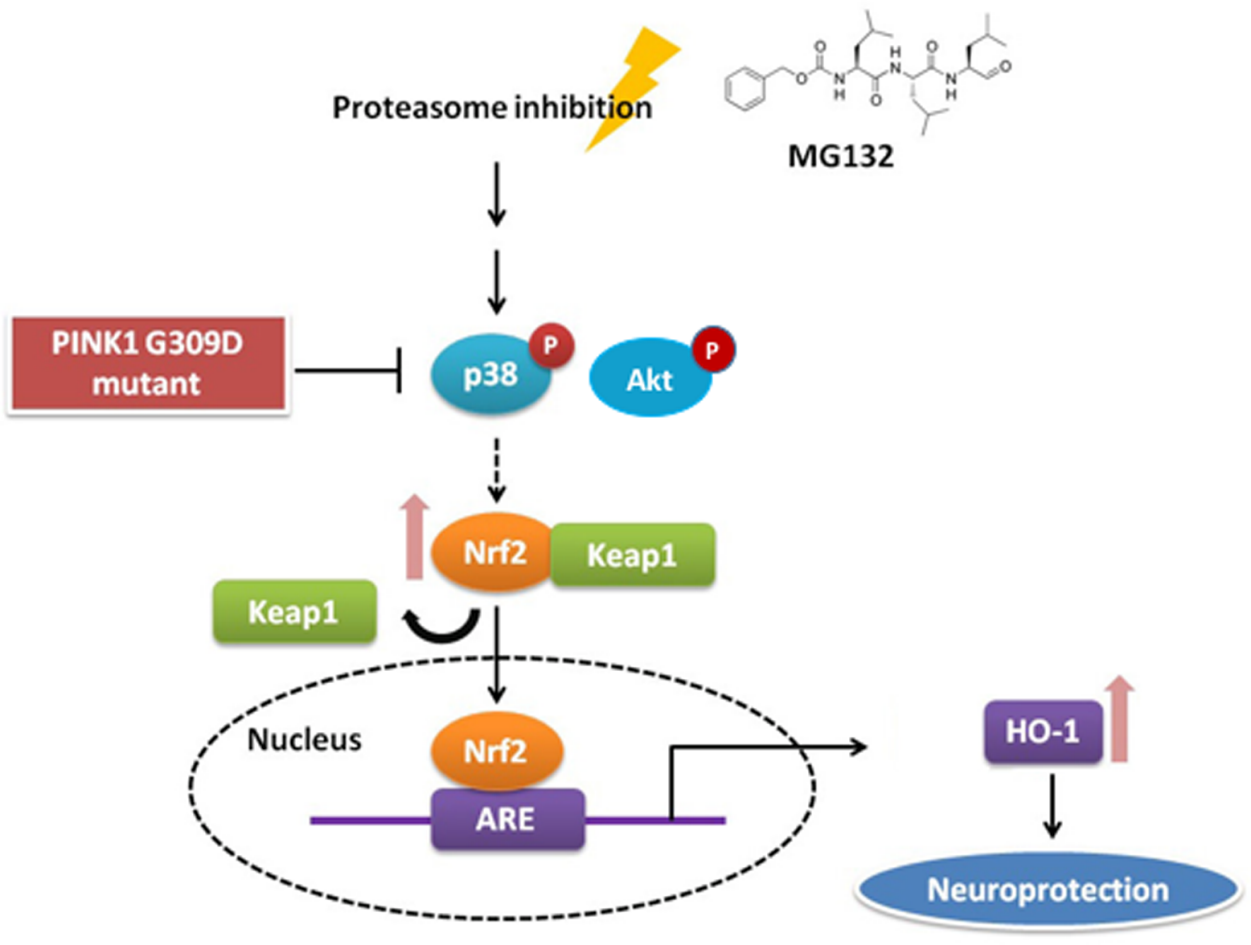
Schematic depiction of the effect of PINK1 G309D mutation on proteasome inhibition-induced cellular response. Under the stress of proteasome inhibition, p38 and Akt are phosphorylated and they activate Nrf2, which then dissociates from Keap1. Activated Nrf2 subsequently translocates into the nucleus and induces the expression of HO-1, which provides a `neuroprotective effect. However, this pathway is abrogated by the PINK1 G309D mutation.

The present study demonstrated that PINK1 G309D mutants may inhibit HO-1 induction under the stress of proteasome inhibition. Upregulation of HO-1 by proteasome inhibition is mediated by the p38-MAPK, Akt and Nrf2 pathways. Moreover, upregulation of HO-1 is also impaired by PINK1 G309D mutation. These results indicate that mutation in *PINK1* reduced the production of neuroprotective enzymes (Fig 7).

Loss-of-function mutations in *PINK1* have been identified to cause early-onset autosomal recessive familial PD [12]. Furthermore, pathogenic mutations in *PINK1*, such as p.K219A, p.G309D, p.L347P, p.D362A, p.D384A, p.G386A, p.G409 V, and p.E417G, are observed at highly conserved positions in the serine-threonine kinase domain [2, 32–34]. Most of mutations are observed in the serine-threonine kinase domain, which suggests that *PINK1* mutations will impair PINK1 kinase activity or substrate recognition and cause PD through a loss of function [35]. The missense mutation G309D was previously reported to impair PINK1 kinase activity, substrate recognition, and further, causes PD through a loss of function [35]. Furthermore, the *PINK1* G309D mutation has been reported to cause a defect in mitochondrial complex 1 activity, thereby leading to an increase in the level of free radicals and extent of of lipid peroxidation [36, 37]. These findings suggested that PINK1 may protect neurons from stress-induced mitochondrial dysfunction and apoptosis, but that protection can be abrogated by the G309D mutation. Although the precise function of PINK1 remains unclear, several studies have suggested that PINK1 may provide a cytoprotective function. In cultured cells, overexpression of *PINK1* confers the resistance to PD-linked neural toxins such as MPTP and rotenone [38, 39]. Moreover, PINK1 expression provides resistance to MPTP-induced dopaminergic neuronal death in mice [2, 39]. Taken together, the investigations on PINK1 suggest a role in mitochondrial dysfunction, protein instability and the derangement of kinase pathways in the pathogenesis of PD; these investigations provide valuable signs regarding the molecular mechanisms of PD [32, 40].

The ubiquitin-proteasome system is a multisubunit complex that degrades various cellular proteins and plays a crucial role in the regulation of cell survival, proliferation, apoptosis, and other critical cellular functions [24, 41]. Several studies have suggested that impairment of the UPS plays a causal role in many neuropathological processes, such as aging, Alzheimer’s disease (AD), PD, amyotrophic lateral sclerosis and Huntington’s disease [1, 42, 43]. When the protein degradation machinery is impaired, these deleterious proteins may accumulate and aggregate to form inclusion bodies, which are pathological hallmarks for several neurodegenerative diseases [5, 44]. Consequently, UPS is essential for cellular functions and survival by participating in the defense against abnormal proteins and providing an effective protein quality control system [44, 45]. Moreover, a decrease in proteasomal function has been observed in the post-mortem brains from patients with AD [46]or PD [47]. Inhibition of the proteasome can result in the toxicity associated with mitochondrial dysfunction and the enhancement of oxidative stress [16, 48]. MG132, the pharmacological inhibitor of proteasome, can produce some features of PD, particularly the formation of proteinaceous inclusion bodies leading to cell death [1, 49, 50]. As a result, we used MG132 as PD-linked neurotoxin in this study.

According to previous studies, HO-1 acts as a protectant against neuronal injury induced by oxidative challenge or excitotoxic injuries. Schipper et al. (1998) demonstrated that the HO-1 expression in the substantia nigra, but not in other brain regions of PD specimens, was 4-fold higher than expression in age-matched controls. In this study, we also found that HO-1 increased in the substantia nigra of MG132-treated rats, thus indicating that HO-1 is a key element for the anti-oxidant defense in the brain and may play a role in neuropathology of PD. Several studies have provided evidence that HO-1 elevation following proteasome inhibition is mediated by Nrf2 and the activation of the p38-MAPK pathway [26, 51]. Nrf2 is a unique stress response transcription factor and is involved in the induction of a variety of genes-encoding proteins, which participate in the detoxification and metabolism of xenobiotics and oxidative stress response [52]. We here demonstrated that MG132 time-dependently increased Nrf2 mRNA and protein levels, indicating that there is a *de novo* synthesis of Nrf2 after proteasome inhibitor treatment. Furthermore, induction of Nrf2 following stress was antagonized by the expression of the recombinant PINK1 G309D mutation. The nuclear translocation of Nrf2 is crucial for the activation of the Nrf2 pathway. The results of nuclear fraction and immunofluorescent staining indicated that the accumulation of Nrf2 was increased in the nucleus following MG132 treatment, but the accumulation of Nrf2 was abrogated by the PINK1 G309D mutation. Akt and p38-MAPK signaling pathways have been reported to be involved in HO-1 expression and Nrf2-dependent transcription [26, 51, 53, 54]. By using specific inhibitors for the Akt and p38 pathway, the involvement of Akt and p38, but not of ERK in MG132-induced HO-1 expression was confirmed. As expected, inhibition of Akt or p38 antagonized MG132-induced Nrf2 nuclear translocation.

In conclusion, we have explored a novel role of PINK1 in the mediation of proteasome inhibitor-induced upregulation of antioxidative enzymes and subsequent neuroprotective action. The pharmacological and genetic approach targeting PINK1 may provide valuable clues to the possible molecular mechanisms underlying PD pathogenesis and represent a feasible strategy for therapeutic treatment of PD.

## Supporting information

S1 Fig Control IgG showed there was no non-specific interaction in immunofluorescence images. Scale bar: 100 μM.
(TIF)

S2 Fig **The inhibitors alone did not affect either (A) HO-1 or (B) Nrf2 basal level expression.** SH-SY5Y cells were pre-treated for 30 min with LY294002 (20 μM), PD98059 (20 μM), SB23580 (10 μM) or N-Acetyl-L-cysteine (NAC) (10 mM), then MG132 (1 μM) was added for 6 hours.
(TIF)
