# Supplementary figures and images for: Antagonism of proteasome inhibitor-induced heme oxygenase-1 expression by PINK1 mutation
Source: PLoS One. 2017 Aug 14;12(8):e0183076. doi: 10.1371/journal.pone.0183076 (PMC5555616; doi:10.1371/journal.pone.0183076)

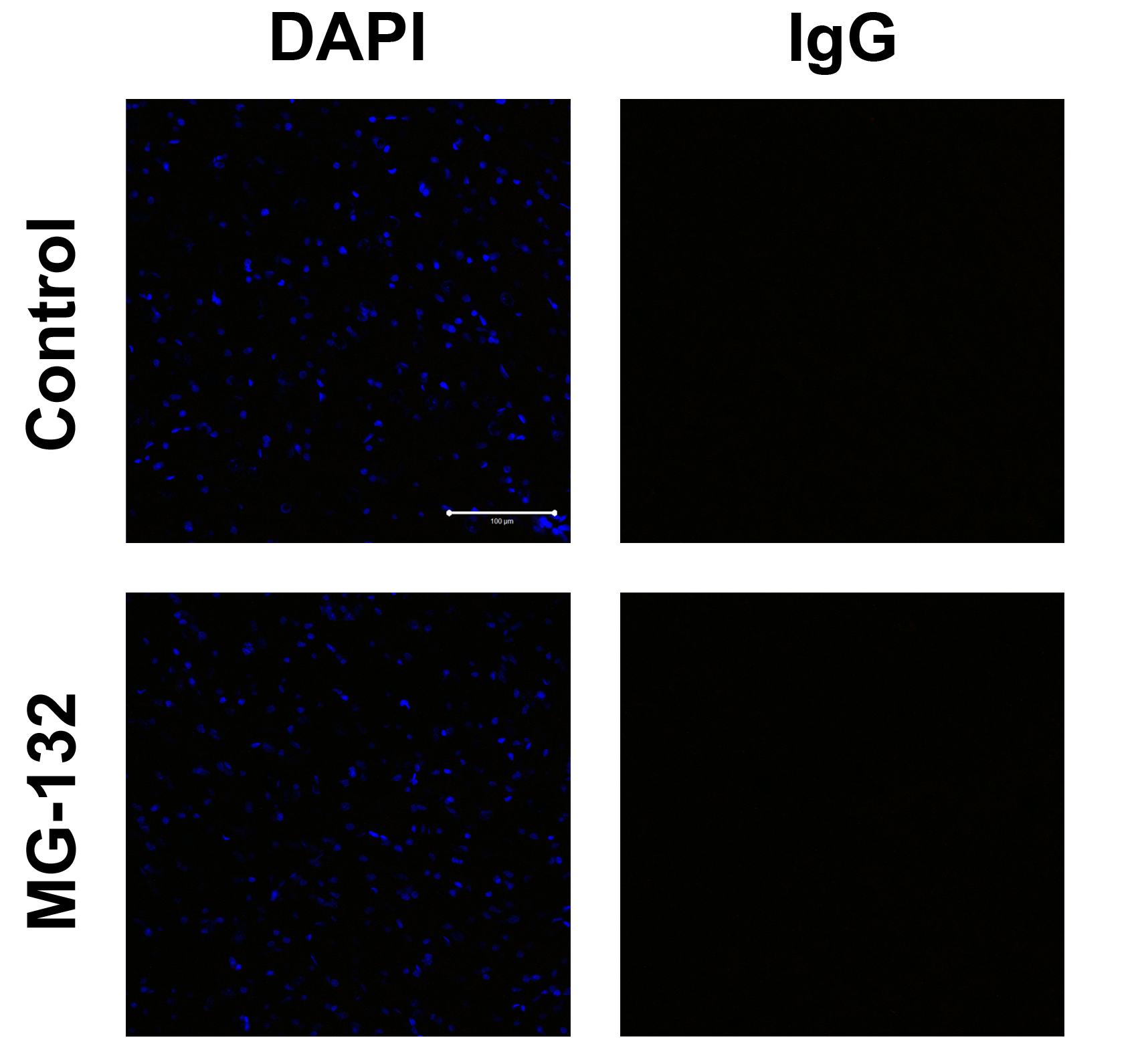

Supplement: S1 Fig — Scale bar: 100 μM. (TIF) [file pone.0183076.s001.tif]

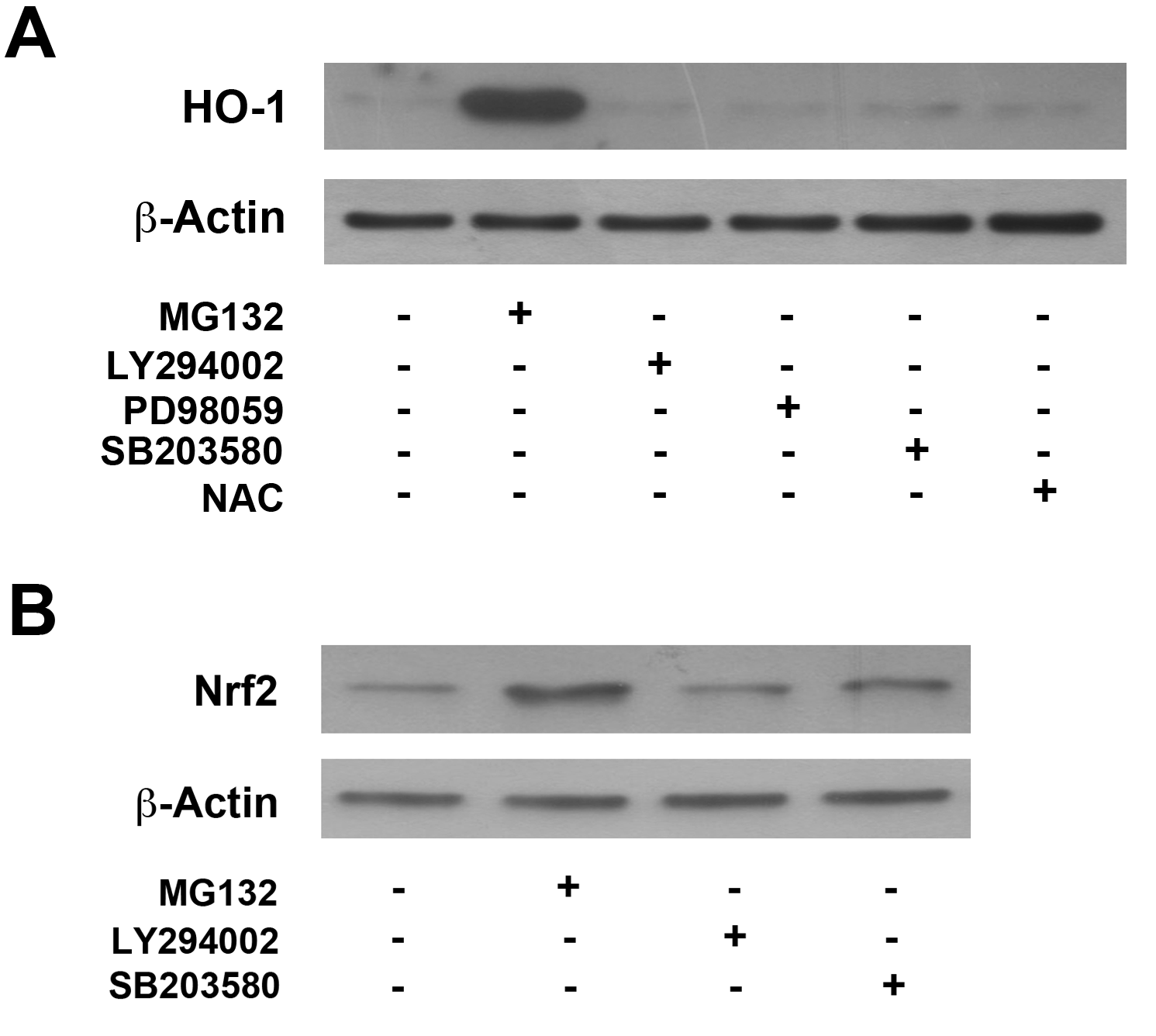

Supplement: S2 Fig — The inhibitors alone did not affect either (A) HO-1 or (B) Nrf2 basal level expression. SH-SY5Y cells were pre-treated for 30 min with LY294002 (20 μM), PD98059 (20 μM), SB23580 (10 μM) or N-Acetyl-L-cysteine (NAC) (10 mM), then MG132 (1 μM) was added for 6 hours. (TIF) [file pone.0183076.s002.tif]
